# Uncovering antimicrobial resistance structures in *Staphylococcus* spp. from companion animals: latent class analysis of isolates from dogs

**DOI:** 10.3389/fvets.2026.1689148

**Published:** 2026-02-05

**Authors:** Alejandra Castro, Brenda Ayzanoa, Vivian Gutierrez, Diego Cuicapuza, Renato Zuñiga, Cusi Ferradas, Guillermo Salvatierra

**Affiliations:** 1 School of Veterinary Medicine, Health Sciences Faculty, Universidad Peruana de Ciencias Aplicadas, Lima, Peru; 2 Department of Microbiology and Immunology, School of Medicine, University of Texas Medical Branch, Galveston, TX, United States; 3 School of Public Health and Administration, Universidad Peruana Cayetano Heredia, Lima, Peru; 4 School of Veterinary Medicine, Universidad Peruana Cayetano Heredia, Lima, Peru

**Keywords:** antimicrobial resistance, companion animals, latent class analysis, Multidrug resistance, Peru, *Staphylococcus* spp.

## Abstract

Antimicrobial resistance (AMR) in companion animals poses therapeutic challenges and potential zoonotic risks. *Staphylococcus* spp., a leading cause of skin and ear infections in dogs and cats, shows high levels of resistance to different families of antibiotics. This study aimed to describe AMR patterns in *Staphylococcus* spp. isolated from companion animals in Lima, Peru, and to identify latent resistance phenotypes among canine isolates using latent class analysis (LCA). We analyzed 2,159 isolates (2,035 from dogs and 124 from cats) collected between 2021 and 2024, assessing resistance to seven antimicrobial classes according to CLSI guidelines. High resistance was found against *β*-lactams (99.7%), followed by sulfonamides (41.6%) and tetracyclines (38.5%), while glycopeptide resistance was rare (0.14%). Overall, MDR prevalence was 49.9%, reaching 52.5% in isolates from dogs. LCA applied to canine isolates identified four latent phenotypes: Phenotype 1 (50.3%) combined *β*-lactam resistance with moderate resistance to macrolides, aminoglycosides, tetracyclines, and sulfonamides; Phenotype 2 (28.2%) showed *β*-lactam resistance with low resistance to other classes; Phenotype 3 (13.6%) included isolates resistant to *β*-lactams and fluoroquinolones; and Phenotype 4 (7.9%) displayed extensive resistance to *β*-lactams, fluoroquinolones, and tetracyclines. LCA revealed hidden resistance structures beyond conventional MDR definitions, identifying subgroups with clinically relevant resistance combinations that may reduce antimicrobial effectiveness. These latent phenotypes have direct therapeutic relevance, as they reveal resistance patterns that may compromise empirical treatment choices in small animal practice. Overall, our findings highlight the need to strengthen AMR surveillance and antimicrobial stewardship within a One Health framework in veterinary medicine.

## 1 Introduction

Antimicrobial resistance (AMR) is a critical global health threat that compromises the effectiveness of treatments in both humans and non-human animals. In animal health, the overuse and misuse of antimicrobials accelerate the emergence and dissemination of resistant pathogens, increasing the likelihood of therapeutic failure and treatment costs (1, 2). Among companion animals, antibiotics are frequently prescribed for dermatological conditions such as pyoderma, wounds, and otitis externa (3, 4), most of which are associated with *Staphylococcus* spp. infections.

*Staphylococcus* spp. is a major opportunistic pathogen and one of the most commonly isolated bacteria from skin and ear infections in dogs and cats (5). While it can be part of the commensal microbiota, certain strains, particularly methicillin-resistant *Staphylococcus* spp. (MRSS), have become increasingly prevalent and challenging to treat (6, 7). These resistant strains undermine the efficacy of first-line antimicrobials, leading to prolonged infections, higher treatment costs, and increased risk of transmission to humans (8). From a One Health perspective, the bidirectional transmission of MRSS between pets and owners further emphasizes the importance of continuous surveillance (2, 9, 10).

Multidrug resistance (MDR), defined as non-susceptibility to at least one agent in three or more antimicrobial classes (11), is particularly concerning. MDR *Staphylococcus* spp. strains have been reported worldwide in companion animals, with resistance most frequently observed against *β*-lactams, macrolides, tetracyclines, fluoroquinolones, and sulfonamides (12–14). In Peru, data on AMR in *Staphylococcus* spp. from companion animals remain scarce, though clinical reports suggest high resistance rates to commonly used antimicrobials, especially within the *β*-lactam and tetracycline classes (15–17).

Traditional surveillance often relies on categorical classifications such as MDR versus non-MDR, which may oversimplify the complexity of resistance profiles. Advanced analytical approaches, such as latent class analysis (LCA), enable the identification of hidden phenotypes by grouping isolates according to their resistance probabilities across multiple antimicrobial classes (18). LCA has been successfully applied in veterinary epidemiology to classify diagnostic patterns and resistance profiles in other pathogens (19, 20); however, its application to *Staphylococcus* spp. in companion animals remains unexplored. A study in Peru analyzing 1,672 isolates collected between 2017 and 2019 from skin and ear samples of dogs and cats in Lima reported an MDR prevalence of 50.4%, with resistance exceeding 99% for penicillin and ampicillin, and high levels for tetracyclines (40.4%) and sulfonamides (43.8%) (21). Although these results are relevant for understanding the burden of antimicrobial resistance, they rely on simplified MDR-based classifications that capture magnitude but fail to reflect the heterogeneity of phenotypic profiles. Traditional MDR-based classifications provide a perspective that may overlook clinically relevant subgroups of resistance. In contrast, LCA can identify hidden phenotypic patterns that reveal combinations of resistances with distinct therapeutic implications, offering a more detailed view of resistance structures (18). This underscores the need for analytical frameworks that reveal latent structures within resistance data. Therefore, this study aimed to describe the antimicrobial resistance profiles of *Staphylococcus* spp. isolated from skin and ear infections in dogs and cats in Lima, Peru, and to compare and identify latent resistance phenotypes among dog isolates using latent class analysis (LCA), as well as to assess factors associated with their presence. This is the first study in veterinary medicine in Peru to apply this approach, and its findings will contribute to a better understanding of resistance patterns and support evidence based antimicrobial stewardship in companion animal clinical.

## 2 Materials and methods

### 2.1 Study design

A retrospective cross-sectional study was conducted using antimicrobial susceptibility data from *Staphylococcus* spp. isolates obtained from microbiological cultures of skin and ear samples collected from companion animals (dogs and cats) treated at a veterinary diagnostic center in Metropolitan Lima, Peru, between July 2021 and December 2024. Each isolate represented a single observation; when multiple isolates were obtained from the same animal during the same clinical episode, only the first isolate was included to avoid duplication. Only confirmed *Staphylococcus* spp. isolates with complete antimicrobial susceptibility testing were analyzed.

### 2.2 Data collection and variables

Data was retrieved from the veterinary diagnostic center’s laboratory information management system. For each isolate, the following variables were extracted: animal species (dog or cat), sex, age (in years), breed (purebred or mixed), sample origin (skin or ear), and antimicrobial susceptibility profile. Antimicrobial susceptibility testing was performed using the disk diffusion method following the Clinical and Laboratory Standards Institute (CLSI) guidelines applicable at the time of testing (CLSI VET01-A4, 2020). Multidrug resistance (MDR) was defined as resistance to at least one antimicrobial agent in three or more different antibiotic classes (11). Twelve antibiotics representing seven major antimicrobial classes were analyzed: *β*-lactams (penicillin, ampicillin, amoxicillin, oxacillin), macrolides (erythromycin), glycopeptides (vancomycin), fluoroquinolones (enrofloxacin, norfloxacin), aminoglycosides (amikacin, gentamicin), tetracyclines (tetracycline), and sulfonamides (sulfamethoxazole/trimethoprim). Isolates lacking complete susceptibility data for these antibiotics were excluded from the analysis. Data were cleaned, validated, and recoded into binary indicators for statistical analysis.

### 2.3 Statistical analysis

Descriptive statistics were used to summarize the demographic and clinical characteristics of the study population. Comparisons between species (dogs vs. cats) were performed using the chi-square test for categorical variables and the Mann–Whitney U test for age, due to its non-normal distribution and heteroscedasticity. The susceptibility profile was determined by measuring the inhibition zone diameters obtained through the disk diffusion test (Kirby-Bauer). The interpretation of these diameters was performed according to the isolates as resistant, intermediate, or susceptible. Intermediate results were grouped with susceptible to ensure a consistent two-category classification and to avoid very small groups that could affect the stability of the LCA model. Thus, susceptibility was dichotomized as resistant versus non-resistant (susceptible and intermediate).

To identify latent resistance phenotypes, a latent class analysis (LCA) model was applied to isolates from dogs only, as the limited number of cat isolates did not meet the minimum sample size required for stable estimation of model parameters. The model was fitted using the Expectation–Maximization (EM) algorithm using random initialization, which iteratively maximizes the likelihood function to estimate class membership probabilities and model parameters. Seven antimicrobial families were included as binary indicators (1 = resistant, 0 = non-resistant) for each isolate. The optimal number of latent classes was selected based on the Bayesian Information Criterion (BIC), combined with clinical interpretability. Posterior class membership probabilities were estimated for each isolate, and isolates were assigned to the class with the highest probability when this exceeded 0.7. All analyses were performed in R (version 4.5.1) within the RStudio environment, using the BayesLCA v1.9 package for latent class analysis. Finally, to compare the distribution of latent resistance phenotypes across host and sample characteristics, we used the Chi-square test for categorical variables and the Kruskal–Wallis test for continuous variables. No correction for multiple testing was applied, as analyses were exploratory in nature. A two-tailed *p*-value <0.05 was considered statistically significant, and 95% confidence intervals (CIs) were reported for relevant estimates.

## 3 Results

### 3.1 Demographic and clinical characteristics of the study population

A total of 2,159 *Staphylococcus* spp. isolates were recovered from companion animals (2,035 from dogs and 124 from cats). The demographic and clinical characteristics of both groups are summarized in Table 1. Sex distribution did not differ significantly between species (*p* = 0.951). Cats were younger than dogs (median 2.0 vs. 4.0 years, *p* < 0.001). Purebred animals were more common among dogs (65.1%), while mixed-breed animals predominated among cats (78.2%; *p* < 0.001). Sample origin also differed significantly between species (*p* < 0.001): most cat isolates were obtained from skin samples (72.6%), whereas in dogs the distribution was more balanced (55.1% skin and 44.9% ear).

**Table 1:** Demographic, clinical, and antimicrobial resistance characteristics of dog and cat *Staphylococcus* spp. isolates (*n* = 2,159).

Characteristics	Total (*n* = 2,159)	Animal species		*p*-value*
		Dogs (*n* = 2035)	Cats (*n* = 124)	
General and clinical				
Sex				
Male	1,187 (55.0)	1,118 (54.9)	55 (44.4)	0.951
Female	972 (45.0)	917 (45.1)	69 (55.6)	
Age (years) ∫	4 (1.5–7.0)	4 (1.5–7)	2 (0.8–5.7)	<0.001**
Breed				
Purebred	1,352 (62.6)	1,325 (65.1)	27 (21.8)	<0.001
Mixed	807 (37.4)	710 (34.9)	97 (78.2)	
Sample origin				
Ear	948 (43.9)	914 (44.9)	34 (27.4)	<0.001
Skin	1,211 (56.1)	1,121 (55.1)	90 (72.6)	
Antimicrobial resistance				
Betalactams				
Penicillin	2,153 (99.7)	2029 (99.7)	124 (100)	>0.999
Ampicillin	2,144 (99.3)	2021 (99.3)	123 (99.2)	0.589
Amoxicillin	142 (6.6)	136 (6.7)	6 (4.8)	0.537
Oxacillin	76 (3.5)	74 (3.6)	2 (1.6)	0.318
Macrolides				
Erythromycin	643 (29.8)	614 (30.2)	29 (23.4)	0.133
Glycopeptides				
Vancomycin	3 (0.14)	3 (0.15)	0 (0.00)	1.000
Fluoroquinolones				
Enrofloxacin	455 (21.1)	424 (20.8)	31 (25.0)	0.322
Norfloxacin	475 (22.0)	439 (21.6)	36 (29.0)	0.066
Aminoglycosides				
Amikacin	124 (5.7)	116 (5.70)	8 (6.5)	0.880
Gentamicin	480 (22.2)	451 (22.2)	29 (23.4)	0.836
Tetracyclines				
Tetracycline	832 (38.5)	788 (38.7)	44 (35.5)	0.532
Sulfonamides				
Sulfamethoxazole/trimethoprim	898 (41.6)	852 (41.9)	46 (37.1)	0.341
MDR phenotype	1,123 (49.9)	1,068 (52.5)	55 (44.4)	0.095

MDR, Multidrug resistance.

*, Chi-square test, **, U Mann–Whitney test, ∫, Median (IQR).

### 3.2 Antimicrobial resistance profiles and comparison between species

**Figure 1:**
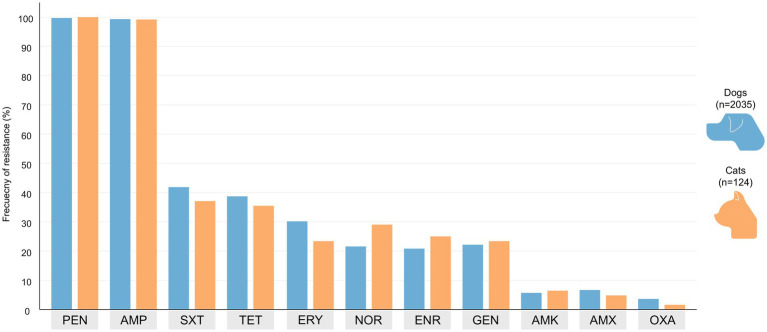
Comparative antimicrobial resistance profiles of *Staphylococcus* spp. isolates from dogs and cats. PEN, Penicillin; AMP, Ampicillin; SXT, Sulfamethoxazole/trimethoprim; TET, Tetracycline; ERY, Erythromycin; NOR, Norfloxacin; ENR, Enrofloxacin; GEN, Gentamicin; AMK, Amikacin; AMX, Amoxicillin; OXA, Oxacillin. The figure shows the proportion of isolates classified as resistant for each antimicrobial agent evaluated in the study. Interpretation of resistance was based on CLSI VET01-A4 (2020) breakpoints.

Across all isolates, phenotypic resistance was observed against all seven antibiotic families evaluated. The highest resistance rates were found for *β*-lactams, with nearly all isolates resistant to penicillin (99.7%) and ampicillin (99.3%). Moderate resistance was observed for tetracycline (38.5%) and sulfamethoxazole/trimethoprim (41.6%). Resistance to erythromycin (29.8%), and fluoroquinolones (21–22%) was also frequent. Only three isolates (0.14%) showed resistance to vancomycin, and resistance to aminoglycosides remained relatively low (5.7% for amikacin and 22.2% for gentamicin) (Figure 1; Table 1). Although MDR was more frequent in canine isolates (52.5%) compared to cat isolates (44.4%), this difference was not statistically significant (*p* = 0.095, Chi-square). No other statistically significant differences in resistance patterns were observed between species (Table 1).

### 3.3 Latent class analysis of resistance phenotypes in canine isolates

**Figure 2:**
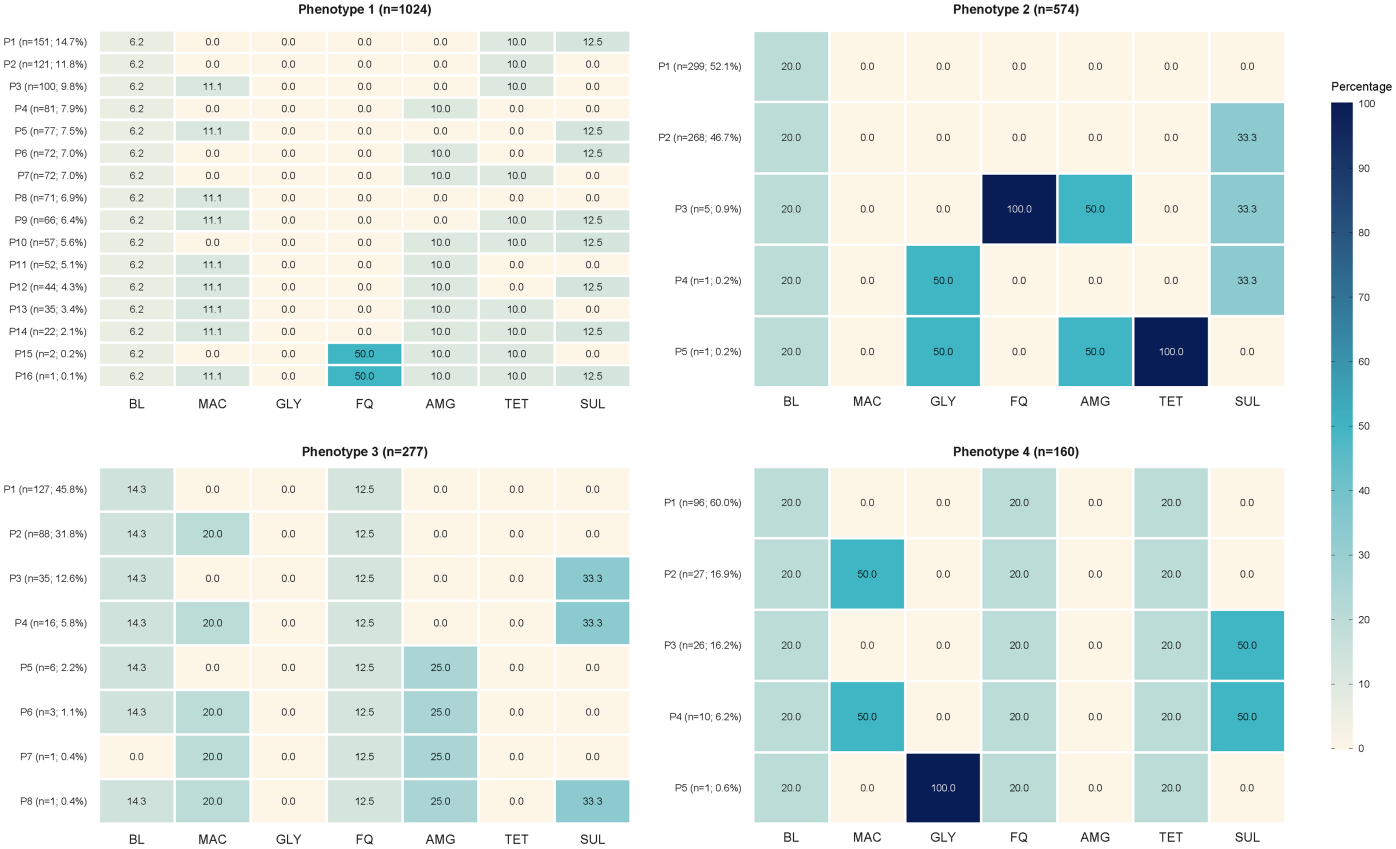
Heatmap of antimicrobial resistance probabilities by latent resistance phenotype among *Staphylococcus* spp. isolates from dogs (*n* = 2,035). The heatmap illustrates the probability of resistance for seven antimicrobial classes across four latent resistance phenotypes derived from latent class analysis. BL, *β*-lactams; MAC, macrolides; GLY, glycopeptides; FQ, fluoroquinolones; AMG, aminoglycosides; TET, tetracyclines; SUL, sulfonamides. Subprofiles within phenotypes: Phenotype 1: P1–P5; Phenotype 2: P6–P8; Phenotype 3: P9–P12; Phenotype 4: P13–P16 (see Supplementary Table 2 for detailed patterns and frequencies).

Latent class analysis (LCA) was conducted on 2,035 *Staphylococcus* spp. isolates obtained from dogs, based on resistance profiles across seven antibiotic families. Comparison of model fit using the BIC indicated that the four-class model (BIC = −12250.06) provided a better balance between fit and parsimony than the model with three classes (BIC = -12216.81), and was therefore selected for interpretation (Supplementary Table 1). Each latent class identified a distinct phenotypic resistance profile (Table 2; Figure 2). Phenotype 1, the most prevalent (*n* = 1,024; 50.3%), was characterized by uniform resistance to *β*-lactams (100%) and moderate resistance to macrolides (45.6%), aminoglycosides (42.7%), tetracyclines (61.1%), and sulfonamides (47.7%), with minimal resistance to fluoroquinolones (0.3%) and no resistance to glycopeptides. Phenotype 2 (*n* = 574; 28.2%) also showed 100% resistance to *β*-lactams but exhibited moderate resistance to sulfonamides (47.8%) and low resistance to the remaining classes, particularly glycopeptides (0.3%) and fluoroquinolones (0.9%), and complete susceptibility to macrolides. Phenotype 3 (*n* = 277; 13.6%) presented a distinct profile with very high resistance to fluoroquinolones (100.0%) and moderate resistance to macrolides (43.6%), but lower resistance to aminoglycosides and tetracyclines. Lastly, Phenotype 4 (*n* = 160; 7.9%) exhibited a multidrug-resistant phenotype, with 100% resistance to *β*-lactams, fluoroquinolones, and tetracyclines, and near-total susceptibility to glycopeptides and aminoglycosides. Across all classes, resistance to glycopeptides remained negligible, and resistance to aminoglycosides was variable but generally lower than that observed for other antibiotic classes. Notably, Phenotype 4 represented the most concerning profile from a clinical perspective, due to its extensive resistance across multiple antimicrobial families. In contrast, Phenotype 2 included isolates with the least resistance beyond *β*-lactams, suggesting a potentially less selective antimicrobial exposure history (Table 3).

**Table 2:** Latent resistance phenotypes (4-class solution) identified by latent class analysis in canine *Staphylococcus* spp. dog isolates (*n* = 2035).

Phenotypes	BL	MAC	GLY*	FQ	AMG	TET	SUL
Phenotype 1 (*n* = 1,024)	100.0%	45.6%	0.0%	0.3%	42.7%	61.1%	47.7%
Phenotype 2 (*n* = 574)	100.0%	0.0%	0.3%	0.9%	1.1%	0.2%	47.8%
Phenotype 3 (*n* = 277)	99.6%	39.5%	0.0%	100.0%	4.1%	0.0%	18.8%
Phenotype 4 (*n* = 160)	100%	23.1%	0.6%	100.0%	0.0%	100.0%	22.4%

BL, beta-lactams; MAC, macrolides; AMG, aminoglycosides; TET, tetracyclines; FQ, fluoroquinolones; SUL, sulfonamides; GLY, glycopeptides.

*Resistance to glycopeptides was observed in 3 isolates overall, but no latent class exhibited a notable pattern for this family due to its very low frequency.

**Table 3:** Distribution of multidrug-resistant (*MDR*) *Staphylococcus* spp. dog isolates by latent resistance phenotype* (*n* = 2035).

Characteristics	Phenotype 1 (*n* = 1,024)	Phenotype 2 (*n* = 574)	Phenotype 3 (*n* = 277)	Phenotype 4 (*n* = 160)	*p*-value*
Sex					0.273**
Male	546 (53.3)	334 (58.2)	148 (53.4)	90 (56.3)	
Female	478 (46.7)	240 (41.8)	129 (46.6)	70 (43.7)	
Age (years)∫	4 (1.7–7)	4 (1.9–7)	3 (1–6)	4 (1.12–7.5)	0.141***
Breed					0.983**
Purebred	671 (65.5)	371 (64.6)	179 (64.6)	104 (65.0)	
Mixed	353 (34.5)	203 (35.4)	98 (35.4)	56 (35.0)	
Sample origin					0.719**
Ear	449 (43.8)	267 (46.5)	128 (46.2)	70 (43.8)	
Skin	575 (56.2)	307 (53.5)	149 (53.8)	90 (56.2)	
MDR					<0.001**
No	273 (26.6)	567 (98.8)	127 (45.8)	0 (0.00)	
Yes	751 (73.4)	7 (1.2)	150 (54.2)	160 (100.0)	

*Results correspond to the four-class latent class model selected based on the Bayesian Information Criterion (BIC).

MDR: Multidrug resistance.

**, Chi-square test, ***, Kruskal-Wallis test.

∫, Median (IQR).

Posterior probabilities for class membership across all observed resistance profiles are presented in Supplementary Table 2. Most profiles exhibited high certainty of assignment to a single latent class, with probabilities exceeding 80%, underscoring the robustness and separability of the four identified phenotypic clusters. A limited number of profiles (five profiles, representing 174 isolates, 8.6% of the dataset) showed moderate classification uncertainty, particularly between Phenotype 1 and 2 or 3, which may reflect overlapping resistance patterns in less common profiles. These findings support the internal validity of the latent class model and its ability to distinguish resistance phenotypes among *Staphylococcus* spp. isolates from canine sources.

## 4 Discussion

In this study, we identified four latent antimicrobial resistance phenotypes among *Staphylococcus* spp. isolates obtained from dog’s skin and ear samples, using latent class analysis (LCA). This approach allowed us to uncover distinct resistance patterns and classify bacterial subpopulations that would not be easily identified using traditional resistance profiling or binary MDR categorizations. The most prevalent class, Phenotype 1, exhibited resistance rates above 30% across most antibiotic families and complete resistance to *β*-lactams, reflecting broad-spectrum exposure or adaptive evolutionary pressure. The remaining phenotypes presented varied resistance profiles, possibly shaped by differential antimicrobial use patterns or ecological pressures in veterinary settings (20). Phenotype 4, although the least frequent, was the most alarming. All isolates within this class showed complete resistance to *β*-lactams, fluoroquinolones, and tetracyclines, thereby qualifying as multidrug-resistant (MDR). This phenotype poses serious clinical and epidemiological concerns, not only due to its therapeutic limitations but also because of the potential for horizontal transmission and persistence within clinical environments (21). The identification of such a class further supports the importance of molecular and phenotypic monitoring to detect emerging MDR phenotypes early and prevent their dissemination (22, 23).

At a broader level, isolates obtained from dogs and cats presented a nearly 100% resistance to *β*-lactams (over 99%), especially to penicillin and ampicillin. These findings are consistent with reports from other regions, where *Staphylococcus* spp. from canine and cat skin or ear infections have shown high levels of *β*-lactam resistance (24, 25). Such resistance is likely driven by the widespread empirical use of these antibiotics in small animal practice, often without prior susceptibility testing (26).

Importantly, the LCA revealed a strong association between resistance phenotypes and MDR status. All isolates classified in Phenotype 4 were MDR, representing 15% of all multidrug-resistant isolates, whereas none in Phenotype 2 exhibited MDR characteristics. These findings suggest that LCA is not only useful for exploratory pattern identification but also effective in isolating clinically relevant subgroups that may be masked by traditional analysis. Notably, no significant associations were observed between class membership and host variables such as age, sex, breed, or sample origin. This suggests that resistance patterns may be more influenced by extrinsic factors such as antimicrobial use practices or environmental exposure rather than intrinsic animal characteristics. This interpretation aligns with prior studies that found no consistent associations between host demographics and methicillin-resistant *Staphylococcus* colonization in veterinary settings (27).

The application of LCA in this context offers significant added value to veterinary epidemiology and antimicrobial surveillance. While LCA has been extensively used in human health research to uncover latent resistance structures and validate diagnostic methods (28, 29), its implementation in veterinary AMR surveillance remains limited. Most prior applications in animal health have focused on mastitis pathogens in livestock or diagnostic test evaluation (30, 31). To our knowledge, this is one of the first studies to apply LCA to *Staphylococcus* spp. isolates from skin and ear infections in dogs. This novel use of LCA highlights its potential as a scalable and flexible tool for identifying resistance phenotypes that can inform empirical therapy and guide antimicrobial stewardship within a One Health framework. Moreover, the heterogeneity observed across phenotypes aligns with previous reports emphasizing the influence of antimicrobial use practices and environmental exposure on resistance patterns in veterinary settings (32), underscoring the importance of continued surveillance and contextualized interpretation of phenotypic data. This study has some limitations that should be acknowledged. First, the analysis was restricted to phenotypic susceptibility data, and no genotypic characterization of resistance mechanisms was performed, which could have provided a deeper understanding of the molecular drivers behind the observed patterns. Second, the sample size of cat isolates was relatively small, limiting the generalizability of findings across species and precluding their inclusion in the latent class analysis. Third, the use of retrospective data from a single diagnostic laboratory in Lima may introduce selection bias, as the isolates may not be fully representative of the broader population of companion animals in Peru. Finally, antimicrobial exposure history and clinical outcomes were not available, which restricted our ability to evaluate risk factors or the clinical relevance of specific resistance phenotypes.

Despite these limitations, our findings provide new insights into the hidden structure of antimicrobial resistance among *Staphylococcus* spp. in companion animals and underscore the value of latent class analysis in unveiling clinically relevant resistance patterns. Recognizing and monitoring these latent phenotypes can improve diagnostic decision-making, guide empirical antimicrobial use, and enhance the effectiveness of veterinary stewardship programs. As veterinary medicine increasingly integrates with One Health approaches, LCA and similar advanced epidemiological methods should be considered essential tools in the fight against antimicrobial resistance.

## Data Availability

The accession number is not provided in the Supplementary Material, and the dataset is not available as currently stated. All relevant data for this study are publicly available from the Figshare repository at https://figshare.com/s/f84262a036c6936f514c.
