# Supplementary material for: Uncovering antimicrobial resistance structures in Staphylococcus spp. from companion animals: latent class analysis of isolates from dogs
Source: Front Vet Sci. 2026 Feb 5;13:1689148. doi: 10.3389/fvets.2026.1689148 (PMC12917887; doi:10.3389/fvets.2026.1689148)
Supplement: Supplementary file 1 [file Data_Sheet_1.docx]

***Supplementary Material***

# Supplementary Tables

## Supplementary Table 1

| **Phenotypes** | **BL** | **MAC** | **GLY*** | **FQ** | **AMG** | **TET** | **SUL** |
| --- | --- | --- | --- | --- | --- | --- | --- |
| **Phenotypes with 4 classes / BIC=-14286.43*** | | | | | | | |
| Phenotype 1 (n=1024) | 100.0% | 43.3% | 0.0% | 1.0% | 34.5% | 49.3% | 46.3% |
| Phenotype 2 (n=574) | 100.0% | 1.8% | 0.0% | 7.1% | 13.6% | 19.7% | 50.5% |
| Phenotype 3 (n=277) | 99.6% | 43.6% | 0.0% | 99.1% | 3.7% | 6.9% | 15.3% |
| Phenotype 4 (n=160) | 100% | 21.0% | 0.0% | 100.0% | 0.0% | 99.6% | 21.3% |
| **Phenotypes with 3 classes / BIC=-14260.08*** | | | | | | | |
| Phenotype 1 (n=967) | 100.0% | 44.4% | 0.0% | 0.0% | 35.9% | 50.4% | 45.6% |
| Phenotype 2 (n=643) | 100.0% | 5.8% | 0.0% | 4.3% | 14.7% | 22.0% | 51.4% |
| Phenotype 3 (n=425) | 99.8% | 34.8% | 0.0% | 97.7% | 3.2% | 37.5% | 18.9% |

**BL = β-lactams, MAC = Macrolides, GLY = Glycopeptides, FQ = Fluoroquinolones, AMG = Aminoglycosides, TET = Tetracyclines, SUL = Sulfonamides**

****Model selection was based on the lowest Bayesian Information Criterion (BIC)***

**Supplementary Table 1.** Comparative results of latent class models with three and four resistance phenotypes based on antimicrobial susceptibility profiles of Staphylococcus spp. isolates from companion dog samples (n = 2035)

- 1. **Supplementary Table 2**

| Phenotype class | Subprofiles | Phenotypes | Class 1 | Class 2 | Class 3 | Class 4 |
| --- | --- | --- | --- | --- | --- | --- |
|  | P1 | 1000010 | 70.14 | 29.80 | 0.05 | 0.01 |
| 1 | P2 | 1000011 | 66.53 | 33.46 | 0.01 | 0.00 |
|  | P3 | 1000100 | 66.49 | 33.46 | 0.04 | 0.00 |
|  | P4 | 1000101 | 62.67 | 37.32 | 0.01 | 0.00 |
|  | P5 | 1000110 | 88.74 | 11.26 | 0.00 | 0.00 |
|  | P6 | 1000111 | 86.94 | 13.06 | 0.00 | 0.00 |
|  | P7 | 1001110 | 38.27 | 38.27 | 23.05 | 0.42 |
|  | P8 | 1100000 | 95.32 | 3.81 | 0.87 | 0.00 |
|  | P9 | 1100001 | 95.31 | 4.51 | 0.18 | 0.00 |
|  | P10 | 1100010 | 98.93 | 1.00 | 0.07 | 0.00 |
|  | P11 | 1100011 | 98.81 | 1.18 | 0.01 | 0.00 |
|  | P12 | 1100100 | 98.76 | 1.18 | 0.07 | 0.00 |
|  | P13 | 1100101 | 98.59 | 1.39 | 0.01 | 0.00 |
|  | P14 | 1100110 | 99.69 | 0.30 | 0.01 | 0.00 |
|  | P15 | 1100111 | 99.64 | 0.36 | 0.00 | 0.00 |
|  | P16 | 1101111 | 86.46 | 2.43 | 11.01 | 0.10 |
| 2 | P1 | 1000000 | 37.12 | 62.55 | 0.33 | 0.00 |
|  | P2 | 1000001 | 33.38 | 66.56 | 0.06 | 0.00 |
|  | P3 | 1001101 | 13.62 | 63.94 | 22.43 | 0.00 |
|  | P4 | 1010001 | 0.00 | 100.00 | 0.00 | 0.00 |
|  | P5 | 1010110 | 0.00 | 100.00 | 0.00 | 0.00 |
| 3 | P1 | 1001000 | 0.81 | 10.82 | 88.11 | 0.26 |
|  | P2 | 1001001 | 2.53 | 39.81 | 57.40 | 0.25 |
|  | P3 | 1001100 | 7.80 | 30.92 | 61.28 | 0.00 |
|  | P4 | 1101000 | 0.90 | 0.28 | 98.71 | 0.10 |
|  | P5 | 1101001 | 4.12 | 1.54 | 94.20 | 0.14 |
|  | P6 | 1101100 | 11.8 | 1.4 | 87.88 | 0.00 |
|  | P7 | 1101101 | 36.06 | 4.2 | 59.92 | 0.00 |
|  | P8 | 0101100 | 0.00 | 0.00 | 100.00 | 0.00 |
| 4 | P1 | 1001010 | 01.06 | 3.55 | 8.79 | 86.60 |
|  | P2 | 1001011 | 3.10 | 12.29 | 5.38 | 79.22 |
|  | P3 | 1011010 | 0.00 | 1.91 | 0.00 | 98.09 |
|  | P4 | 1101010 | 2.65 | 0.21 | 22.15 | 74.99 |
|  | P5 | 1101011 | 8.55 | 0.80 | 14.97 | 75.68 |

**Supplementary Table 2.** Distribution of resistance subprofiles (P1–P16) among Staphylococcus spp. isolates from dogs (n = 2,035) and their correspondence to latent resistance phenotypes identified by latent class analysis. The table lists binary resistance patterns (coded as BL-MAC-GLY-FQ-AMG-TET-SUL), the number of isolates within each subprofile, and their relative frequency within each phenotype. Subprofiles were grouped into four major latent resistance phenotypes: Phenotype 1 (broad resistance including β-lactams and moderate resistance to other classes), Phenotype 2 (β-lactam resistance only), Phenotype 3 (β-lactams plus fluoroquinolones), and Phenotype 4 (extensive resistance to β-lactams, fluoroquinolones, and tetracyclines).
